# Assessment of Secondary Sarcomas Among Patients With Cancer of the Abdomen or Pelvis Who Received Combinations of Surgery, Radiation, and Chemotherapy vs Surgery Alone

**DOI:** 10.1001/jamanetworkopen.2020.13929

**Published:** 2020-10-02

**Authors:** Amanda E. Hird, Diana E. Magee, Rano Matta, Refik Saskin, Erind Dvorani, Girish S. Kulkarni, Ronald Kodama, Sender Herschorn, Steven A. Narod, Robert K. Nam

**Affiliations:** 1 Division of Urology, Sunnybrook Health Science Centre, University of Toronto, Toronto, Ontario, Canada; 2 Institute of Health Policy, Management, and Evaluation, University of Toronto, Toronto, Ontario, Canada; 3 Division of Urology, University Health Network, University of Toronto, Toronto, Ontario, Canada; 4 Institute for Clinical Evaluative Sciences, Toronto, Ontario, Canada; 5 Women’s College Research Institute, Women’s College Hospital, Toronto, Ontario, Canada; 6 Dalla Lana School of Public Health, University of Toronto, Toronto, Ontario, Canada

## Abstract

**Importance:**

The association of radiation and chemotherapy with the development of secondary sarcoma is known, but the contemporary risk has not been well characterized for patients with cancers of the abdomen and pelvis.

**Objective:**

To compare the risk of secondary sarcoma among patients treated with combinations of surgery, radiation, or chemotherapy with patients treated with surgery alone and the general population.

**Design, Setting, and Participants:**

This population-based cohort study included 173 580 patients in Ontario, Canada, with nonmetastatic cancer of the prostate, bladder, colon, rectum or anus, cervix, uterus, or testis. Patients were enrolled from January 1, 2002, to January 31, 2017. Data analysis was conducted from March 1, 2019, to January 31, 2020.

**Exposures:**

Treatment combinations of radiation, chemotherapy, and surgery.

**Main Outcome and Measures:**

Diagnosis of sarcoma based on histologic codes from the Ontario Cancer Registry. Time to sarcoma was compared using a cause-specific proportional hazard model.

**Results:**

Of 173 580 patients, most were men (125 080 [72.1%]), and the largest group was aged between 60 and 69 years (58 346 [33.6%]). Most patients had genitourinary cancer (86 235 [51.4%]) or colorectal cancer (69 241 [39.9%]). Overall, 64 301 (37.1%) received surgery alone, 51 220 (29.5%) received radiation alone, 15 624 (9.0%) were treated with radiation and chemotherapy, 15 252 (8.8%) received radiation with surgery, and 11 822 (6.8%) received all 3 treatments. A total of 332 patients (0.2%) had sarcomas develop during a median (interquartile range) follow-up of 5.7 (2.2-8.9) years. The incidence of sarcoma was 0.3% among those who underwent radiation alone (138 of 51 220) and radiation with chemotherapy (40 of 15 624), 0.2% among those who received radiation and surgery (36 of 15 252) and all 3 modalities (25 of 11 822), and 0.1% among those who received surgery with chemotherapy (13 of 14 861) and surgery alone (80 of 64 801). Compared with a reference group of patients who had surgery alone, the greatest risk of sarcoma was found among patients who underwent a combination of radiation and chemotherapy (cause-specific relative hazard [csRH], 4.07; 95% CI, 2.75-6.01; *P* < .001), followed by patients who had radiation alone (csRH, 2.35; 95% CI, 1.77-3.12; *P* < .001), radiation with surgery (csRH, 2.33; 95% CI, 1.57-3.46; *P* < .001), and all 3 modalities (csRH, 2.27; 95% CI, 1.44-3.58; *P* < .001). In the general population, 7987 events occurred during 46 554 803 person-years (17.2 events per 100 000 person-years). The standardized incidence ratio for sarcoma among patients treated with radiation compared with the general population was 2.41 (95% CI, 1.57-3.69; 41.3 events per 100 000 person-years). The annual number of cases of sarcoma increased from 2009 (15 per 100 000 persons) to 2016 (32 per 100 000 persons), but the annual rate did not change during the study period.

**Conclusions and Relevance:**

In this cohort study, patients treated with radiation or chemotherapy for abdominopelvic cancers had an increased rate of sarcoma. Although the absolute rate is low, patients and physicians should be aware of this increased risk of developing sarcoma.

## Key Points

**Question:**

What is the rate of secondary sarcoma among patients with nonmetastatic cancer of the prostate, bladder, colon, rectum or anus, cervix, uterus, or testis who were treated with combinations of surgery, radiation, or chemotherapy compared with patients treated with surgery alone and with the general population?

**Findings:**

In this cohort study of 173 580 patients, the rate of secondary sarcoma among patients treated with radiotherapy was increased compared with patients who had surgery alone and with the general population. The risk was highest among those who received both chemotherapy and radiotherapy.

**Meaning:**

This study provides further evidence to support the association of radiotherapy with secondary sarcoma; the finding of an increase in risk with combination radiotherapy and chemotherapy, in particular, merits further study.

## Teaser

This cohort study compares the risk of secondary sarcoma among patients treated with combinations of surgery, radiation, and chemotherapy with patients treated with surgery alone and with the general population.

## Introduction

Approximately 60% of all patients diagnosed with cancer will undergo radiotherapy for the treatment of their disease.^1^ Radiation treatment has been associated with the development of secondary malignant neoplasms.^2,3,4^ However, because of the effects of common exposures, including smoking and other confounding factors, the causal relationship remains unclear. In contrast, the development of secondary sarcoma after radiation treatment has been well established and there are minimal effects of confounding variables.^5,6,7,8,9,10,11,12,13,14^ Chemotherapy treatment may also contribute to the development of secondary cancers, particularly hematologic malignant neoplasms,^15,16,17,18,19,20,21^ but the relationship between chemotherapy use and sarcoma risk has been poorly studied.

Several cohort studies have analyzed the risk of secondary cancers from various individual primary cancer sites^22,23,24^ but have not used the development of sarcoma as the primary end point and have been underpowered^22,25^ owing to its rare incidence. Also, no studies have evaluated the association of chemotherapy with the risk of developing a secondary sarcoma across a broad group of primary abdominopelvic cancers.

To evaluate the association of radiation and chemotherapy treatment with secondary sarcoma, we examined all patients diagnosed with cancers of the abdomen and pelvis from a large population-based cohort. We compared the relative rate of secondary sarcoma among patients treated with combinations of surgery, radiation, or chemotherapy with patients treated with surgery alone and with the general population. We hypothesized that the relative rate of sarcoma would be higher for patients treated with radiation or chemotherapy compared with patients treated with surgery alone.

## Methods

### Study Subjects

This was a retrospective cohort study using population-based administrative data examining the risk of secondary sarcoma among patients diagnosed with nonmetastatic cancer of the prostate, cervix, bladder, colon, rectum or anus, uterus, or testis between January 1, 2002, and January 31, 2017. Data analysis was conducted between March 1, 2019, and January 31, 2020. Patients were identified using the Ontario Cancer Registry (OCR). Patients were excluded if they were found to have metastatic disease at the time of diagnosis or if they had a pre-existing invasive cancer diagnosis (except for patients with nonmelanomatous skin cancers). To include only those patients actively receiving medical care in Ontario during the study interval, we excluded individuals who died as well as those who emigrated prior to the index date.

Data were extracted through the Institute of Clinical and Evaluative Sciences (ICES) Data-Analytic Services. ICES is an independent, nonprofit research institute whose legal status under Ontario’s health information privacy law allows it to collect and analyze health care and demographic data without consent for health system evaluation and improvement. This study was designed and conducted according to the Strengthening the Reporting of Observational Studies in Epidemiology (STROBE) reporting guideline.^26^ The study was approved by the Sunnybrook research ethics board.

### Primary Outcome and Exposure Variables

The primary outcome was the rate of any sarcoma that developed at least 6 months after the date of primary treatment,^1^ identified using cancer histology codes from the OCR. The OCR is known to capture more than 95% of all malignant neoplasms based on *International Classification of Disease* (*ICD*) coding.^27^ Sarcoma diagnosis was captured using histology codes. No studies have validated the capture of sarcomas based on histologic codes in adult patients. However, among pediatric patients with sarcoma, validation studies have shown high sensitivity and specificity for soft tissue and extraosseous sarcomas (0.82 and 0.99, respectively) and malignant bone tumors (0.92 and 0.99, respectively).^28^ For the purposes of the standardized incidence ratio (SIR) calculation, sarcoma diagnosis was based on *ICD* codes (eAppendix 1 in the Supplement).

The key exposure variables were the combination of modalities used to treat the primary cancer, including surgery, radiation, or chemotherapy. All medical procedures in Ontario are reimbursed by a government-operated health insurance system, the Ontario Health Insurance Plan (OHIP). OHIP fee codes are listed for specific procedures. We used OHIP fee codes and Canadian Classification of Health Intervention (CCI) codes to identify patients who underwent abdominopelvic surgery, and fee codes X310, X311, X312, and X313 (planning codes for radiation) or X323, X324, X325, or X305 (intracavitary codes for radiation) and corresponding CCI codes to identify patients who underwent abdominopelvic radiation^22^ (eAppendix 2 in the Supplement). The index date was defined as the first primary treatment date. Validation studies of the OHIP database have shown 88% to 96% agreement with medical record abstraction for accurate capture of procedural data.^29^

Chemotherapy use was collected using a combination of OHIP, CCI, and *International Statistical Classification of Diseases and Related Health Problems, Tenth Revision *(*ICD*-*10*) codes (eAppendix 2 in the Supplement). Intent of chemotherapy is not recorded in administrative data. To identify perioperative and periradiotherapy chemotherapy use, any chemotherapy code that occurred 16 weeks before or after the date of primary treatment was captured.

We also examined baseline covariates, including age, sex, John Hopkins Aggregate Disease Group (ADG) comorbidity score, income quintile, and rural vs urban residence. A look-back window of 2 years was used to ascertain ADG score. All data sets were linked using unique encoded identifiers and analyzed at ICES.

### Statistical Analysis

Descriptive characteristics were compared among primary treatment groups. The treatment groups included surgery alone, radiation alone, and combinations of surgery, radiation, and chemotherapy. Cause-specific hazard models are appropriate for addressing epidemiological questions of etiology.^30,31^ We used a cause-specific proportional hazard model to determine the association between primary treatment and secondary sarcoma, accounting for the competing risk of death (all-cause). Univariable and multivariable cause-specific relative hazards were reported as a measure of the association of a covariate with the relative instantaneous hazard rate of sarcoma among individuals who survived to that time point.^30^ We adjusted for age, income quintile, urban vs rural residence, and ADG comorbidity score in our multivariable analysis.

Multicollinearity was assessed within the model. Assumptions were verified. Clustering was accounted for at the level of the treating institution, using a generalized estimating equation. Cause-specific relative hazard ratios (csRHs) with 95% CIs are reported. Analysis was completed on the overall cohort and stratified by primary cancer site. A 2-tailed *P* < .05 was used to indicate statistical significance. All analyses were performed using SAS version 9.4 (SAS Institute).

The SIR for the development of sarcoma was calculated as the ratio of the observed number of sarcoma cases divided by the age-stratified and sex-stratified expected number of sarcoma cases from the Ontario population per 100 000 persons^32^ separated by primary exposure group (surgery alone and radiation alone) owing to few events. Because of the potential lag time that is required for secondary cancers to develop after radiation treatment, with the reported range between 5 and 10 years,^22,33,34^ we conducted an analysis of a subgroup of patients who developed secondary sarcomas after a minimum of 7 years after the date of primary treatment.

## Results

Of the 173 580 patients in the cohort, most patients were men (125 080 [72.1%]), and the largest group was aged between 60 and 69 years (58 346 [33.6%]) (Table 1). Most patients had genitourinary cancer (86 235 [51.4%]) or colorectal cancer (69 241 [39.9%]). The most common primary malignant neoplasms were from the prostate (80 081 [46.1%]) followed by colon (43 349 [25.0%]), and rectum or anus (25 892 [14.9%]). Nearly one-quarter of patients received chemotherapy (42 307 of 173 580 [24.4%]); 64 801 (37.3%) underwent surgery alone, 14 861 (8.6%) underwent surgery with chemotherapy, 51 220 (29.5%) underwent radiation alone, 15 624 (9.0%) were treated with radiation and chemotherapy, 15 252 (8.8%) received radiation with surgery, and 11 822 (6.8%) received all 3 treatments. There were statistically significant differences between our exposure groups based on age (eg, aged 18-49 years: surgery alone, 3554 [5.5%]; surgery and chemotherapy, 2017 [13.6%]; radiation alone, 1487 [2.9%]; radiation and chemotherapy, 2626 [16.8%]; radiation and surgery, 574 [3.8%]; all 3 treatments, 1643 [13.9%]; *P* < .001), primary cancer site (eg, prostate cancer: surgery alone, 28 953 [44.7%]; surgery and chemotherapy, 342 [2.3%]; radiation alone, 37 364 [73.0%]; radiation and chemotherapy, 2663 [17.0%]; radiation and surgery, 10 125 [66.4%]; all 3 treatments, 634 [5.4%]; *P* = .001), comorbidity score (eg, ADG score ≥13: surgery alone, 6594 [10.2%]; surgery and chemotherapy, 1347 [9.1%]; radiation alone, 8164 [15.9%]; radiation and chemotherapy, 3027 [19.4%]; radiation and surgery, 2509 [16.5%]; all 3 treatments, 2145 [18.1%]; *P* < .001), income quintile (eg, lowest income quintile: surgery alone, 10 994 [17.0%]; surgery and chemotherapy, 2608 [17.6%]; radiation alone, 8965 [17.5%], radiation and chemotherapy, 3169 [20.3%]; radiation and surgery, 2367 [15.5%]; all 3 treatments, 2185 [18.5%]; *P* < .001), and geographic region (eg, rural residence: surgery alone, 9701 [15.0%;] surgery and chemotherapy, 2201 [14.8%]; radiation alone, 7248 [14.2%]; radiation and chemotherapy, 2238 [14.3%]; radiation and surgery, 2223 [14.6%]; all 3 treatments, 1821 [15.4%]; *P* < .001). Compared with women, men were more likely to have received surgery alone (17 451 [26.9%] vs 47 350 [73.1%]), radiation alone (9577 [18.7%] vs 41 643 [81.3%], radiation with surgery (2137 [14.0%] vs 13 115 [86.0%]), and all 3 treatment modalities (48 500 [27.9%] vs 125 080 [72.1%]) whereas women were more likely to have received radiation with chemotherapy (8473 [54.2%] vs 7151 [45.8%]). Compared with patients in the surgery-alone group, patients who received radiation tended to be older (aged ≥70 years: 29 800 of 79 662 [37.4%] vs 42 248 of 93 918 [45.0%]), have a higher comorbidity score (ADG ≥9: 34 305 of 79 662 [43.1%] vs 52 418 of 93 918 [55.8%]), and have received chemotherapy (14 861 of 79 662 [18.7%] vs 27 446 of 93 918 [29.2%]).

**Table 1.** Demographic Variables by Exposure Group

Variable	No. (%)							*P* value
	Surgery alone (n = 64 801)	Surgery and chemotherapy (n = 14 861)	Radiation alone (n = 51 220)	Radiation and chemotherapy (n = 15 624)	Radiation and surgery (n = 15 252)	Radiation, surgery, and chemotherapy (n = 11 822)	Overall (N = 173 580)	
Age, y								
18-49	3554 (5.5)	2017 (13.6)	1487 (2.9)	2626 (16.8)	574 (3.8)	1643 (13.9)	11 901 (6.9)	<.001
50-59	13 244 (20.4)	3183 (21.4)	5489 (10.7)	3459 (22.1)	2986 (19.6)	2924 (24.7)	31 285 (18.0)	
60-69	23 081 (35.6)	4783 (32.2)	15 548 (30.4)	4438 (28.4)	6663 (43.7)	3833 (32.4)	58 346 (33.6)	
70-79	14 693 (22.7)	3901 (26.3)	21 114 (41.2)	3686 (23.6)	3675 (24.1)	2820 (23.9)	49 889 (28.7)	
≥80	10 229 (15.8)	977 (6.6)	7582 (14.8)	1415 (9.1)	1354 (8.9)	602 (5.1)	22 159 (12.8)	
Sex								
Women	17 451 (26.9)	6497 (43.7)	9577 (18.7)	8473 (54.2)	2137 (14.0)	4365 (36.9)	48 500 (27.9)	<.001
Men	47 350 (73.1)	8364 (56.3)	41 643 (81.3)	7151 (45.8)	13 115 (86.0)	7457 (63.1)	125 080 (72.1)	
Primary cancer								
Prostate	28 953 (44.7)	342 (2.3)	37 364 (73.0)	2663 (17.0)	10 125 (66.4)	634 (5.4)	80 081 (46.1)	.001
Cervix	962 (1.5)	43 (0.3)	920 (1.8)	2707 (17.3)	147 (1.0)	350 (3.0)	5129 (2.9)	
Bladder	2166 (3.3)	822 (5.5)	2303 (4.5)	1478 (9.5)	548 (3.6)	465 (3.9)	7782 (4.5)	
Colon	26 027 (40.2)	11 132 (74.9)	1177 (2.3)	1437 (9.2)	1863 (12.2)	1713 (14.5)	43 349 (25.0)	
Rectum or anus	6366 (9.8)	2146 (14.4)	2115 (4.1)	4391 (28.1)	2396 (15.7)	8478 (71.7)	25 892 (14.9)	
Uterus	153 (0.2)	66 (0.4)	6660 (13.0)	2766 (17.7)	162 (1.1)	168 (1.4)	9975 (5.8)	
Testis	174 (0.3)	310 (2.1)	681 (1.3)	182 (1.2)	11 (0.1)	14 (0.1)	1372 (0.8)	
ADG score								
0-4	6581 (10.2)	1949 (13.1)	2917 (5.7)	807 (5.2)	971 (6.4)	675 (5.7)	13 900 (8.0)	<.001
5-8	30 108 (46.5)	6719 (45.2)	20 497 (40.0)	5290 (33.9)	5997 (39.3)	4346 (36.8)	72 957 (42.0)	
9-12	21 518 (33.2)	4846 (32.6)	19 642 (38.4)	6500 (41.6)	5775 (37.9)	4656 (39.4)	62 937 (36.3)	
≥13	6594 (10.2)	1347 (9.1)	8164 (15.9)	3027 (19.4)	2509 (16.5)	2145 (18.1)	23 786 (13.7)	
Income quintile								
1, lowest	10 994 (17.0)	2608 (17.6)	8965 (17.5)	3169 (20.3)	2367 (15.5)	2185 (18.5)	30 288 (17.5)	<.001
2	12 892 (19.9)	2969 (20.0)	10 238 (20.0)	3354 (21.5)	2879 (18.9)	2366 (20.0)	34 698 (20.0)	
3	12 898 (19.9)	3081 (20.7)	10 147 (19.8)	3100 (19.8)	3064 (20.1)	2360 (20.0)	34 650 (20.0)	
4	13 291 (20.5)	3062 (20.6)	10 447 (20.4)	2961 (19.0)	3260 (21.4)	2486 (21.0)	35 507 (20.5)	
5, highest	14 726 (22.7)	3141 (21.1)	11 423 (22.3)	3040 (19.5)	3682 (24.1)	2425 (20.5)	38 437 (22.1)	
Rural								
No	55 100 (85.0)	12 660 (85.2)	43 972 (85.9)	13 386 (85.7)	13 029 (85.4)	10 001 (84.6)	148 148 (85.4)	<.001
Yes	9701 (15.0)	2201 (14.8)	7248 (14.2)	2238 (14.3)	2223 (14.6)	1821 (15.4)	25 432 (14.7)	
LHIN								
1	3720 (5.7)	784 (5.3)	3243 (6.3)	822 (5.3)	738 (4.8)	643 (5.4)	9950 (5.7)	<.001
2	6290 (9.7)	1231 (8.3)	3934 (7.7)	1407 (9.0)	1356 (8.9)	1218 (10.3)	15 436 (8.9)	
3	3554 (5.5)	722 (4.9)	2560 (5.0)	809 (5.2)	976 (6.4)	685 (5.8)	9306 (5.4)	
4	8007 (12.4)	1609 (10.8)	6353 (12.4)	1813 (11.6)	1696 (11.1)	1308 (11.1)	20 786 (12.0)	
5	3143 (4.9)	730 (4.9)	2356 (4.6)	729 (4.7)	787 (5.2)	487 (4.1)	8232 (4.7)	
6	4510 (7.0)	1068 (7.2)	3891 (7.6)	1043 (6.7)	1165 (7.6)	793 (6.7)	12 470 (7.2)	
7	4477 (6.9)	1143 (7.7)	3958 (7.7)	1407 (9.0)	1078 (7.1)	774 (6.6)	12 837 (7.4)	
8	7166 (11.1)	1827 (12.3)	5588 (10.9)	1677 (10.7)	1735 (11.4)	1306 (11.1)	19 299 (11.1)	
9	8067 (12.5)	1805 (12.2)	5183 (10.1)	1734 (11.1)	1775 (11.6)	1424 (12.1)	19 988 (11.5)	
10	2607 (4.0)	688 (4.6)	2515 (4.9)	717 (4.6)	570 (3.7)	522 (4.4)	7619 (4.4)	
11	5879 (9.1)	1467 (9.9)	6039 (11.8)	1665 (10.7)	1640 (10.8)	1117 (9.5)	17 807 (10.3)	
12	2737 (4.2)	641 (4.3)	1723 (3.4)	598 (3.8)	738 (4.8)	457 (3.9)	6822 (3.9)	
13	3475 (5.4)	848 (5.7)	3005 (5.9)	868 (5.6)	1356 (8.9)	810 (6.9)	9798 (5.6)	
14	1169 (1.8)	298 (2.0)	872 (1.7)	335 (2.1)	976 (6.4)	278 (2.4)	3230 (1.9)	

Abbreviations: ADG, Aggregate Disease Group; LHIN, Local Health Integration Network.

Overall, 332 patients (0.2%) were diagnosed with sarcoma during the study period (Table 2). The most common tumors were leiomyosarcoma (36 [10.8%]), gastrointestinal stromal sarcoma (31 [9.3%]), liposarcoma (30 [9.0%]), and carcinosarcoma (28 [8.4%]) (eAppendix 3 in the Supplement). The median (interquartile range) time from primary treatment to the diagnosis of sarcoma was 5.7 (2.2-8.9) years (Table 2). The incidence of sarcoma was 0.3% among those who underwent radiation alone (138 of 51 220) and radiation with chemotherapy (40 of 15 624), 0.2% among those who received radiation and surgery (36 of 15 252) and all 3 modalities (25 of 11 822), and 0.1% among those who received surgery with chemotherapy (13 of 14 861) and surgery alone (80 of 64 801).

**Table 2.** Study Outcomes by Exposure Group

Outcome	Surgery alone (n = 64 801)	Surgery and chemotherapy (n = 14 861)	Radiation alone (n = 51 220)	Radiation and chemotherapy (n = 15 624)	Radiation and surgery (n = 15 252)	Radiation, surgery, and chemotherapy (n = 11 822)	*P* value
Sarcoma, No. (%)							
No	64 721 (99.9)	14 848 (99.9)	51 082 (99.7)	15 584 (99.7)	15 216 (99.8)	11 797 (99.8)	<.001
Yes	80 (0.1)	13 (0.1)	138 (0.3)	40 (0.3)	36 (0.2)	25 (0.2)	
Death during study period, No. (%)							
No	46 433 (71.7)	8810 (59.3)	30 649 (59.8)	6134 (39.3)	10 453 (68.5)	5936 (50.2)	<.001
Yes	18 368 (28.4)	6051 (40.7)	20 571 (40.2)	9490 (60.7)	4799 (31.5)	5886 (49.8)	
Follow-up, median (IQR), y	7.3 (3.8-11.3)	5.2 (2.6-9.7)	6.3 (2.9-10.4)	2.7 (0.8-6.5)	5.9 (3.0-9.4)	4.9 (1.9-9.3)	<.001
Time to sarcoma, median (IQR), y	5.5 (2.9-8.7)	6.0 (1.3-10.1)	6.4 (2.9-9.4)	5.2 (2.5-8.3)	5.0 (2.6-8.9)	5.8 (4.6-8.1)	.81

Abbreviations: IQR, interquartile range.

Compared with the baseline group of patients who had surgery alone, patients who had radiation treatment alone or in combination with surgery or chemotherapy had an increased risk of developing sarcoma. On univariable and multivariable analysis, the risk of developing sarcoma was highest among patients who underwent a combination of radiation and chemotherapy (adjusted csRH, 4.07; 95% CI, 2.75-6.01; *P* < .001), compared with patients who had surgery alone (Table 3). This was followed by patients who had radiation alone (adjusted csRH, 2.35; 95% CI, 1.77-3.12; *P* < .001), radiation with surgery (adjusted csRH, 2.33; 95% CI, 1.57-3.46; *P* < .001), and radiation with surgery and chemotherapy (adjusted csRH, 2.27; 95% CI, 1.44-3.58; *P* < .001) (Table 3). There was no difference in the relative rate of sarcoma among those treated with surgery and chemotherapy compared with surgery alone (adjusted csRH, 0.91; 95% CI, 0.50-1.63; *P* = .74). Age was not a significant factor (Table 3). When accounting for clustering at the level of the institution, there was no significant change in our parameter estimates.

**Table 3.** Univariable and Multivariable Cause-Specific Hazard Model for Time to Sarcoma

Variable	Univariable analysis		Multivariable analysis	
	csRH (95% CI)	*P* value	csRH (95% CI)	*P* value
Exposure				
Surgery alone	1 [Reference]	NA	1 [Reference]	NA
Surgery and chemotherapy	0.89 (0.49-1.60)	.69	0.91 (0.50-1.63)	.74
Radiation alone	2.49 (1.89-3.28)	<.001	2.35 (1.77-3.12)	<.001
Radiation and chemotherapy	4.10 (2.80-5.99)	<.001	4.07 (2.75-6.01)	<.001
Radiation and surgery	2.41 (1.62-3.57)	<.001	2.33 (1.57-3.46)	<.001
Radiation, surgery, and chemotherapy	2.31 (1.47-3.62)	<.001	2.27 (1.44-3.58)	<.001
Age group, y				
≤49	1 [Reference]	NA	1 [Reference]	NA
50-59	0.89 (0.56-1.44)	.64	1.02 (0.63-1.65)	.93
60-69	1.06 (0.69-1.64)	.79	1.17 (0.75-1.84)	.49
70-79	1.27 (0.82-1.99)	.29	1.24 (0.78-1.97)	.36
≥80	1.12 (0.62-2.02)	.70	1.29 (0.70-2.35)	.42
ADG score	1.07 (1.03-1.11)	<.001	1.05 (1.01-1.09)	.01
Income quintile				
1, lowest	1 [Reference]	NA	1 [Reference]	NA
2	0.73 (0.50-1.06)	.10	0.74 (0.51-1.08)	.12
3	0.93 (0.65-1.33)	.69	0.96 (0.67-1.36)	.80
4	1.09 (0.77-1.52)	.64	1.13 (0.81-1.59)	.48
5, highest	0.92 (0.65-1.29)	.61	0.97 (0.68-1.36)	.84
Residence				
Urban	1 [Reference]	NA	1 [Reference]	NA
Rural	1.19 (0.89-1.58)	.24	1.22 (0.92-1.63)	.17

Abbreviations: ADG, Aggregate Disease Group; csRH, cause-specific relative hazard; NA, not applicable.

To account for the overlapping association of sex with primary cancer site, we stratified our analysis by primary cancer site (eAppendix 4 in the Supplement). Compared with surgery alone, we found the association between radiation and the risk of sarcoma was highest for patients with colon cancer (radiation alone: adjusted csRH, 9.52; 95% CI, 2.84-31.90; *P* < .001; radiation with surgery: adjusted csRH, 8.22; 95% CI, 3.08-21.90; *P* < .001; radiation with surgery and chemotherapy: adjusted csRH, 9.03; 95% CI, 3.03-26.93; *P* < .001).

Patients with prostate cancer who received radiation also had a significantly increased relative rate of sarcoma compared with those who received surgery alone (radiation alone: adjusted csRH, 2.22; 95% CI, 1.50-3.29; *P* < .001; radiation with surgery: adjusted csRH, 2.09; 95% CI, 1.26-3.45; *P* = .004). However, the rate was not significantly different among those treated with radiation with chemotherapy (adjusted csRH, 2.04; 95% CI, 0.49-8.50; *P* = .33) compared with patients who received surgery alone (eAppendix 4 in the Supplement).

The relative rate of sarcoma was significantly higher among patients with cancer of the rectum or anus treated with radiation and chemotherapy (adjusted csRH, 3.67; 95% CI, 1.48-9.09; *P* = .005) but not among those treated with combination radiation, surgery, and chemotherapy (adjusted csRH, 2.08; 95% CI, 0.90-4.82; *P* = .09) compared with surgery alone (eAppendix 4 in the Supplement). We could not complete subgroup analyses on patients with cancer of the cervix, bladder, uterus, or testis because there were too few sarcoma events.

When we restricted our analysis to patients who developed sarcoma beyond a period of 7 years from the date of primary treatment, the adjusted csRH was 3.38 (95% CI, 2.11-5.42; *P* < .001) among those who received radiation alone, 4.97 (95% CI, 2.50-9.86; *P* < .001) among those who received radiation with chemotherapy, 3.70 (95% CI, 1.95-7.03; *P* < .001) among those who received radiation and surgery, and 2.83 (95% CI, 1.32-6.09; *P* < .001) among those who received all 3 treatments compared with patients who received surgery alone. There was no difference in the relative rate of sarcoma among those treated with surgery and chemotherapy compared with surgery alone (adjusted csRH, 1.15; 95% CI, 0.44-3.01; *P* = .77).

We further examined the rates of sarcoma by year of diagnosis following the 7-year lag period separated by primary exposure group (surgery vs radiation). The crude annual incidence of sarcoma was constant among patients who underwent surgery and the general population but appeared to be increasing among patients who received radiation (15 cases per 100 000 persons in 2009 vs 32 cases per 100 000 persons in 2016) (Figure). The observed increase in the incidence of sarcoma in the radiation group appeared to be associated with increasing exposure time given that the time to sarcoma from the index date was longer for patients diagnosed in the last 3 years of the study period when the observed increase was most pronounced (eAppendix 5 in the Supplement). We also grouped patients by index date (2002-2006, 2007-2011, 2012-2016) and did not find any difference in the actuarial cumulative incidence rate of sarcoma (log-rank *P* = .62).

**Figure.**
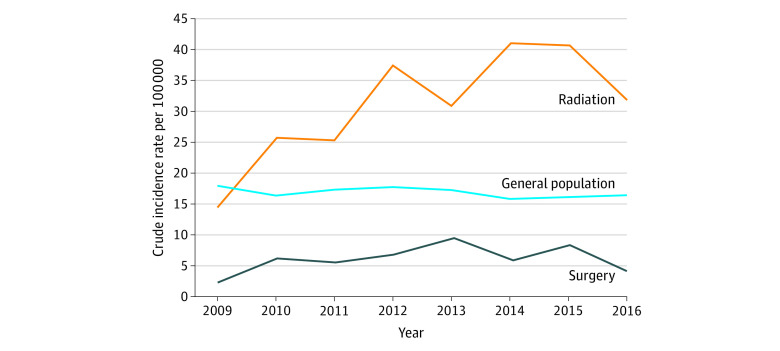
Crude Incidence of Sarcoma per 100 000 Persons by Primary Treatment and for the General Population of Ontario

In the general population, 7987 events occurred during 46 554 803 person-years (17.2 events per 100 000 person-years). The SIR was calculated for patients who had radiation alone and surgery alone to keep each group homogenous when comparing with the general population during the study period. In the radiation-alone group, 110 events were observed during 299 725 person-years (standardized rate 41.3 events per 100 000 person-years) compared with an expected 17.2 events per 100 000 person-years. Patients who received radiation had a significantly increased risk of sarcoma compared with the general population (SIR, 2.41; 95% CI, 1.57-3.69; *P* < .001). In the surgery-alone group, 34 events were observed during 412 780 person-years (standardized rate, 4.6 events per 100 000 person-years). Patients who received surgery had a decreased risk of sarcoma compared with the general population (SIR, 0.27; 95% CI, 0.16-0.44; *P* < .001) (Table 4).

**Table 4.** Age-Standardized and Sex-Standardized Incidence Ratios for Risk of Developing a Sarcoma for Patients in the Radiation-Alone and Surgery-Alone Groups, Compared With the General Population of Ontario, 2003-2016

Population	Observed events	Person-years	Crude rate, per 100 000 person-years	Standardized rate per 100 000 person-years	Risk ratio (95% CI)	*P* value
General population	7987	46 554 803	17.2	17.2	NA	NA
Radiation alone	110	299 725	36.7	41.3	2.41 (1.57-3.69)	<.001
Surgery alone	34	412 780	8.2	4.6	0.27 (0.16-0.44)	<.001

Abbreviation: NA, not applicable.

## Discussion

Among a large population-based cohort of 173 580 patients, we found an increased rate of secondary sarcoma among patients treated for abdominopelvic cancer with radiation with or without surgery and chemotherapy compared with patients who had surgery alone. Patients treated for primary colon cancer had the highest risk. Compared with the general population, patients treated with radiation alone had more than twice the rate of secondary sarcoma. The number of sarcoma cases appeared to be increasing over time.

To our knowledge, this is the largest and most comprehensive series to estimate the risk of secondary sarcoma in a large group of adult patients treated for multiple primary abdominopelvic cancers. Large population-based studies in the United States among men with prostate cancer^23^ and women with endometrial cancer^2^ have demonstrated an increased risk of a second solid tumor of any type among those treated with radiation. Similar findings were reported in a population-based data set of men with prostate cancer in British Columbia^3^ and Ontario.^22^ However, these studies focused on all secondary malignant neoplasms and not specifically on sarcoma. They also included patients with only 1 primary disease site, which limited the sample size.

Virtanen et al^35^ conducted a large, retrospective population-based study in Finland investigating the risk of sarcoma in patients with breast, cervical, uterine, ovarian, lung, prostate, and rectal cancer as well as Hodgkin and non-Hodgkin lymphoma treated with radiation, chemotherapy, or neither. The adjusted relative rates of sarcoma among patients treated with radiotherapy alone and radiotherapy with chemotherapy compared with those who received neither were not significant (RR = 1.5; 95% CI, 0.9-2.6 and RR = 0.8; 95% CI, 0.1-5.8, respectively). The likely reason they did not demonstrate an association is because of the selection of study participants, which included patients with cancers that are often associated with poor survival (specifically lung and ovarian cancers) and failure to exclude patients with metastatic disease at enrollment. We chose to study a group of patients with nonmetastatic, primary abdominopelvic cancers with better cancer-specific survival rates. We also accounted for death as a competing risk on regression analysis.

Patients who were treated with radiation had an increased rate of sarcoma compared with the general population. However, patients who received surgery alone had a lower rate of sarcoma compared with the general population. Patients selected for surgery may have been healthier than the general population. A study comparing the rate of diagnosis of any secondary cancer among patients with primary prostate cancer also reported a lower SIR among patients treated with surgery compared with the general population; this was most pronounced in older patients.^22^ Likewise, observational studies comparing all-cause mortality rates among patients with cancer vs general population controls have shown lower mortality rates among patients treated with surgery.^36^ The potential effect of selection bias and unmeasured confounding may explain this decrease in risk among patients who underwent surgery.

Multivariable regression analysis may generate a more accurate comparison of the secondary sarcoma rate relative to other treatment groups by accounting for comorbidity, income quintile, and geographic region. In our study, we found that the hazard of sarcoma among the group that received all 3 treatment modalities was lower than that among the group that received radiation and chemotherapy. It is possible that the patients undergoing all 3 treatment modalities received lower doses of radiation and chemotherapy to improve tolerability. As chemotherapeutic agent and specific details about radiation (ie, dose and modality) are not well captured in administrative data, it remains to be seen whether these differences in relative rate are associated with specific details about the treatment regimen.

It is also of great interest that the rate of sarcoma is increasing over time. We are unaware of any change in diagnostic imaging or protocols for sarcoma during the study period. We hypothesize that this increase in the rate of sarcoma is secondary to patients living longer after radiation exposure. Because the number of sarcoma cases did not increase in the surgery group, this increase may reflect improvements in overall cancer-specific survival after radiation treatment, allowing for more sarcomas to develop in patients with radiation exposure.^37^ It may also be possible that the emergence of complex radiation treatment modalities may place patients at an increased risk of developing secondary cancers.^38^

The strengths of this analysis include the population level of this data and the large sample size of more than 170 000 patients across multiple primary cancer sites and at multiple centers in Ontario. We had few missing data points overall, excluding less than 0.5% of patients from our analysis. This study is at low risk of misclassification of the primary outcome given that validation studies have revealed high capture rates in the OCR.^27,28^

### Limitations

There are several limitations to our study. If patients moved outside of Ontario during the study period, we were not able to capture events. With all administrative studies, there is potential for misclassification bias. There is potential for misclassification of our primary exposure group given that radiation use was primarily based on the billing of a radiotherapy planning code and does not necessarily indicate treatment. This would bias our findings toward the null hypothesis. Similarly, we found that approximately 4.5% of patients with prostate cancer received systemic chemotherapy within 16 weeks of prostate cancer or prostate radiation, which is higher than what would be expected. Possible explanations for this include the use of systemic therapy for early disease recurrence and progression, clinical trial–associated neoadjuvant chemotherapy, or off-label use for patients with locally advanced disease. Only a medical chart review would determine the indication for chemotherapy for these patients, which was not be feasible for this study.

Although we were able to control for many of the differences among groups in our analysis, there may be differences with respect to other unmeasured confounding factors, including specific details about the cancer itself, radiation dosage, chemotherapy agent, and genetic predisposition, that cannot be accounted for. By limiting chemotherapy exposure to treatment occurring within 16 weeks of primary therapy, our estimate on the association between this variable and the risk of secondary sarcoma may be underestimated due to misclassification of patients who received perioperative chemotherapy outside of this window. Also, the association between cumulative number of chemotherapy cycles and subsequent cancer risk was outside of the scope of the current study. Further studies are needed to explore the association of radiation dosage, radiation route, and cumulative chemotherapy dose with secondary sarcoma risk in this population.

Unfortunately, due to few events, we were unable to calculate the SIR for some exposure groups. When we separated these events by year, age group, and sex, there was considerable imprecision around our SIR estimates. Because of limitations of the OCR database, while we were able to ascertain histological information about the secondary sarcomas that developed during the study period, we were unable to capture specific information about the location of the secondary tumors.

## Conclusions

This cohort study found an increased rate of secondary sarcoma among patients treated for abdominopelvic cancer with radiation therapy compared with patients who had surgery and with the general population. The strongest association was seen among patients treated for primary colon cancer and for those who received both radiotherapy and chemotherapy. The incidence of sarcoma among patients treated with radiotherapy appeared to be increasing over time, potentially because of longer survival from the treatment of their initial cancer.

## Notes

Supplement. **eAppendix 1. **Primary and Secondary Outcome Definitions **eAppendix 2. **Classification of Exposure and Other Covariates **eAppendix 3. **Sarcoma Histology **eAppendix 4. **Primary Cancer Site–Stratified Multivariable Cause-Specific Proportional Hazard Model, Time to Sarcoma
