# Supplementary material for: Assessment of Secondary Sarcomas Among Patients With Cancer of the Abdomen or Pelvis Who Received Combinations of Surgery, Radiation, and Chemotherapy vs Surgery Alone
Source: JAMA Netw Open. 2020 Oct 2;3(10):e2013929. doi: 10.1001/jamanetworkopen.2020.13929 (PMC7532387; doi:10.1001/jamanetworkopen.2020.13929)
Supplement: Supplement. — eAppendix 1. Primary and Secondary Outcome Definitions eAppendix 2. Classification of Exposure and Other Covariates eAppendix 3. Sarcoma Histology eAppendix 4. Primary Cancer Site–Stratified Multivariable Cause-Specific Proportional Hazard Model, Time to Sarcoma [file jamanetwopen-e2013929-s001.pdf]

Supplemental Online Content

Hird AE, Magee DE, Matta R, et al. Assessment of secondary sarcomas among patients with cancer of the abdomen or pelvis who received combinations of surgery, radiation, and chemotherapy vs surgery alone. *JAMA Netw Open*. 2020;3(10):e2013929.
doi:10.1001/jamanetworkopen.2020.13929

eAppendix 1. Primary and Secondary Outcome Definitions

eAppendix 2. Classification of Exposure and Other Covariates

eAppendix 3. Sarcoma Histology

eAppendix 4. Primary Cancer Site–Stratified Multivariable Cause-Specific Proportional Hazard Model, Time to Sarcoma

This supplemental material has been provided by the authors to give readers additional information about their work.

© 2020 Hird AE et al. *JAMA Network Open.*

eAppendix 1. Primary and Secondary Outcome Definitions

<u>Primary: Sarcoma diagnosis based on ICD diagnostic codes from the Ontario Cancer Registry in order to compute standardized incidence ratio (SIR)</u>

Any one of the following:

C380 (malignant neoplasm of the pericardium)

C470–479 (malignant neoplasm of peripheral nerves and ANS)

C490–499 (malignant neoplasm of other connective and soft tissue)

C400–409 (malignant neoplasm of bone and articular cartilage of limbs)

C410–419 (malignant neoplasm of bone and articular cartilage of other and unspecified sites)

© 2020 Hird AE et al. *JAMA Network Open.*

eAppendix 2. Classification of Exposure and Other Covariates

Classification of Exposure

Radiotherapy:

Any one of the following:

OHIP radiation planning codes: X310 [simple], X311 [intermediate], X312 [complex], X313 [IMRT/3D CRT]

OR

Intracavitary radiotherapy application: X323, X324, X325, or X305

OR

CCI radiotherapy codes:

- Cervical [1RN27^^]
- Prostate [1QT27^^]
- Uterus [1RM27^^]
- Colon [1NM27^^]
- Rectum/anus [1NQ27^^ or 1NT27^^]
- Bladder [1PM27^^]
- Testis [1MG27JA]

OR

CCI brachytherapy codes:

- Cervical [1RN26^^]
- Prostate [1QT26^^]
- Uterus [1RM26^^]
- Colon [none]
- Rectum/anus [1NQ26^^ or 1NT26^^]
- Bladder [1PM26^^]
- Testis [none]

Surgery:

Any one of the following:

OHIP surgery codes:

- Cervical [S763 or S762]
- Prostate [S651 or S653]
- Uterus [S763]
- Colon [S166, S167, S168, S169, S172, or S171]
- Rectum/anus [S167, S170, S171, S213, S214, S215, or S216]
- Bladder [S482 or S483 or S490 or S484 or S485 or S453 or S440 or S441]
- Testis [S598 or S590]

OR

CCI surgery codes:

- Cervical [1RM91^^ or 1RN91^^]
- Prostate [1QT91^^]
- Uterus [1RM91^^]

© 2020 Hird AE et al. *JAMA Network Open.*

- Colon [1NM87DF, 1NM87RN, 1NM87DE, 1NM87RD, 1NM87DN, 1NM87RE, 1NM87DX, 1NM87DY, 1NM87TG, 1NM87TF, 1NM87PN, 1NM89^^, or 1NM91^^]
- Rectum/anus [1NQ87LA, 1NQ87RD, 1NQ87SF, 1NQ87TF, 1NQ87DA, 1NQ87DE, 1NQ87DF, 1NQ87DX, 1NQ87PN, or 1NQ89^^]
- Bladder [1PM91LA or 1PM92^^]
- Testis [1MG87^^, 1MG89^^]

Other Covariates

Chemotherapy (yes/no) occurring either 16 weeks BEFORE or 16 weeks AFTER index date (date of primary treatment)

OHIP: G381, G388, G281, G339, G345, G359, G382, or G390, OR
CCP: 13.55 injection or infusion of cancer chemotherapeutic substance or antineoplastic agent, OR
CCI: 1ZZ35, OR
ICD–10 Z511 encounter for antineoplastic chemotherapy and immunotherapy, ICD–10 Z542 convalescence following chemotherapy

© 2020 Hird AE et al. *JAMA Network Open.*

eAppendix 3. Sarcoma Histology

Total number of sarcomas=332. Most common sarcoma histologies are presented. Due to ICES privacy policy, cells with small counts must be suppressed.

Code	Histology	Number of cases
88903	Leiomyosarcoma	36
89363	Gastrointestinal stromal sarcoma	31
89803	Carcinosarcoma	28
88513	Liposarcoma	15
88583	Dedifferentiated liposarcoma	15
88023	Giant cell sarcoma	14
80333	Sarcomatoid carcinoma	12
80033	Sarcoma NOS	8
92203	Chondrosarcoma	8
91203	Hemangiosarcoma/angiosarcoma	7
88323	Dermatofibrosarcoma	7
88113	Fibromyosarcoma	7

© 2020 Hird AE et al. *JAMA Network Open.*

eAppendix 4. Primary Cancer Site–Stratified Multivariable Cause-Specific Proportional Hazard Model, Time to Sarcoma

a) Prostate cancer

Variable	Multivariable analysis		
	csRH	95% CI	p–value
Exposure			
Surgery only	1.0		
Surgery and chemotherapy	—	—	—
Radiation only	2.22	1.50–3.29	*<0.001*
Radiation and chemotherapy	2.04	0.49–8.50	0.33
Radiation and surgery	2.09	1.26–3.45	*0.004*
Radiation, surgery and chemotherapy	—	—	—
Age group (years)			
≤49	1.0		
50–59	2.41	0.33–17.78	0.39
60–69	3.13	0.43–22.53	0.26
70–79	3.55	0.49–25.85	0.21
80+	1.15	0.10–12.90	0.91
ADG score	1.04	0.99–1.09	0.17
Income quintile			
1	1.0		
2	0.90	0.52–1.55	0.70
3	1.03	0.61–1.73	0.93
4	1.33	0.81–2.17	0.26
5	0.93	0.56–1.55	0.79
Residence			
Urban	1.0		
Rural	1.62	1.13–2.34	*0.010*

ADG: John Hopkins aggregate disease group comorbidity score; csRH: cause-specific relative hazard

© 2020 Hird AE et al. *JAMA Network Open.*

b) Colon cancer

Variable	Multivariable analysis		
	**csRH**	**95% CI**	**p–value**
Exposure			
Surgery only	1.0		
Surgery and chemotherapy	0.68	0.30–1.53	0.35
Radiation only	9.52	2.84–31.90	*<0.001*
Radiation and chemotherapy	—	—	—
Radiation and surgery	8.22	3.08–21.90	*<0.001*
Radiation, surgery and chemotherapy	9.03	3.03–26.93	*<0.001*
Age group (years)			
≤49	1.0		
50–59	0.54	0.15–2.03	0.36
60–69	1.06	0.35–3.22	0.92
70–79	0.75	0.24–2.37	0.62
80+	1.37	0.41–4.55	0.61
Gender			
Male	1.0		
Female	1.51	0.84–2.73	0.17
ADG score	0.93	0.85–1.02	0.13
Income quintile (reference: 1)			
1	1.0		
2	0.68	0.25–1.82	0.44
3	1.22	0.51–2.90	0.65
4	0.68	0.25–1.83	0.45
5	1.10	0.46–2.62	0.83
Residence			
Urban	1.0		
Rural	0.64	0.25–1.62	0.34

ADG: John Hopkins aggregate disease group comorbidity score; csRH: cause-specific relative hazard

© 2020 Hird AE et al. *JAMA Network Open.*

c) Rectal/anal cancer

Variable	Multivariable analysis		
	csRH	95% CI	p–value
Exposure			
Surgery only	1.0		
Surgery and chemotherapy	2.47	0.80–7.60	0.12
Radiation only	1.20	0.15–9.73	0.87
Radiation and chemotherapy	3.67	1.48–9.09	*0.005*
Radiation and surgery	2.52	0.82–7.73	0.11
Radiation, surgery and chemotherapy	2.08	0.90–4.82	0.09
Age group (years)			
≤49	1.0		
50–59	1.09	0.34–3.53	0.89
60–69	2.25	0.77–6.58	0.14
70–79	1.91	0.62–5.88	0.26
80+	2.27	0.58–8.82	0.24
Gender			
Male	1.0		
Female	0.70	0.40–1.21	0.20
ADG score	1.15	1.06–1.25	*0.001*
Income quintile			
1	1.0		
2	1.00	0.41–2.46	0.99
3	0.82	0.32–2.12	0.68
4	1.58	0.70–3.58	0.27
5	0.99	0.40–2.44	0.99
Residence			
Urban	1.0		
Rural	1.09	0.51–2.31	0.83

ADG: John Hopkins aggregate disease group comorbidity score; csRH: cause-specific relative hazard

© 2020 Hird AE et al. *JAMA Network Open.*

APPENDIX 5. Time to secondary sarcoma, by year of diagnosis.

Sarcoma Year	Group	Mean Index Year	SD	Median Index Year	IQR	Median Days to Sarcoma	IQR
2009	Radiation	2006.60	0.55	2007	2006–2007	688	572–934
	Surgery	2003.00	—	2003	—	2046	—
2010	Radiation	2005.10	1.73	2005	2004–2007	1958	1034–2330
	Surgery	2005.33	1.53	2005	2004–2007	1820	946–2207
2011	Radiation	2006.55	2.73	2007	2005–2008	1506	958–2031
	Surgery	2007.67	1.53	2008	2006–2009	1051	559–1655
2012	Radiation	2006.44	3.09	2007	2004–2009	1908	1175–3040
	Surgery	2008.50	1.00	2009	2008–2009	1104	1048–1450
2013	Radiation	2007.88	3.32	2008	2006–2011	1985	653–2725
	Surgery	2009.17	3.76	2011	2006–2012	852	403–2524
2014	Radiation	2008.87	2.82	2009	2006–2011	1894	1120–2831
	Surgery	2005.50	2.89	2006	2004–2008	3009	2328–3839
2015	Radiation	2007.46	2.77	2007	2006–2010	2948	1756–3311
	Surgery	2007.67	3.78	2008	2005–2009	2795	2094–3737
2016	Radiation	2008.35	4.08	2007	2005–2013	3258	1347–3906
	Surgery	2007.00	1.41	2007	2006–2008	3348	2951–3745

SD: standard deviation; IQR: interquartile range

© 2020 Hird AE et al. *JAMA Network Open.*
